# Reconfigurable and scalable 2,4-and 6-channel plasmonics demultiplexer utilizing symmetrical rectangular resonators containing silver nano-rod defects with FDTD method

**DOI:** 10.1038/s41598-021-93167-y

**Published:** 2021-07-01

**Authors:** Shiva Khani, Ali Farmani, Ali Mir

**Affiliations:** 1 Faculty of Electrical and Computer Engineering, Semnan University, Semnan, Iran; 2 School of Electrical and Computer Engineering, Lorestan University, Khoramabad, Iran

**Keywords:** Optical materials and structures, Optical physics

## Abstract

Reconfigurable and scalable plasmonics demultiplexers have attracted increasing attention due to its potential applications in the nanophotonics. Therefore, here, a novel method to design compact plasmonic wavelength demultiplexers (DEMUXes) is proposed. The designed structures (two, four, and six-channel DEMUXes) consist of symmetrical rectangular resonators (RRs) incorporating metal nano-rod defects (NRDs). In the designed structures, the RRs are laterally coupled to metal–insulator-metal (MIM) waveguides. The wavelengths of the output channels depend on the numbers and radii of the metal NRDs in the RRs. The results obtained from various device geometries, with either a single or multiple output ports, are performed utilizing a single structure, showing real reconfigurability. The finite-difference time-domain (FDTD) method is used for the numerical investigation of the proposed structures. The metal and insulator used for the realization of the proposed DEMUXes are silver and air, respectively. The silver’s permittivity is characterized by the well-known Drude model. The basic plasmonic filter which is used to design plasmonic DEMUXes is a single-mode filter. A single-mode filter is easier to cope with in circuits with higher complexity such as DEMUXes. Also, different structural parameters of the basic filter are swept and their effects on the filter’s frequency response are presented, to provide a better physical insight. Taking into account the compact sizes of the proposed DEMUXes (considering the six-channel DEMUX), they can be used in integrated optical circuits for optical communication purposes.

## Introduction

Surface plasmon polaritons (SPPs) are the electromagnetic waves that confine at the boundary of two metal and insulator materials with different signs of dielectric constants^1–3^. SPPs are attracting tremendous consideration due to their outstanding abilities such as overcoming the diffraction limit^4,5^ and manipulating light at a sub-wavelength scale^6^. As a result, they are suitable candidates for the realization of highly integrated optical circuits. Various structures have been proposed based on SPPs such as plasmonic filters^7,8^, sensors^9–12^, directional couplers^13,14^, splitters^15^, demultiplexers (DEMUXes)^16,17^, logic gates^18–20^, modulators^21,22^, switches^23–26^ and so on. One of the most important devices in optical communication is DEMUX structures. Wavelength division multiplexing systems need optical DEMUXes. Accordingly, a huge amount of studies based on various structures and resonator configurations have been directed on the realization of DEMUX structures.

Nano-disk resonators are one of the most practical resonator types in plasmonic structures due to their simplicity in the design/ fabrication process and tunable resonance frequencies. Therefore, various plasmonic DEMUXes have been designed based on such resonators^16,27,28^. In^28^, to achieve a three-channel plasmonic DEMUX, several nano-disk resonators with different radii have been used. In^27^, in addition to using nano-disk resonators with different radii, the refractive indexes of these resonators have also been changed. Nano-disk resonators usually create dual or multi-mode transmission spectra which is not desirable for designing DEMUX devices. Accordingly, in^16^, nano-disk resonators are coupled to parenthesis-shaped adjunctions to obtain single mode transmission spectra.

Another method that has been used in^29^ is using hexagonal resonators to design plasmonic DEMUXes. Although such resonators have a more difficult fabrication process, they have a better coupling ability with metal–insulator-metal (MIM) waveguides compared to nano-disk resonators. Using stub resonators is another technique, which has been used to design optical DEMUXes^30^. One of the main advantages of such structures is that they can be analyzed using the transmission line method which is faster than the conventional finite-difference time-domain (FDTD) method.

Another type of resonator used in many different DEMUX structures is ring-shaped resonators. Given this, various DEMUX configurations using such resonators including circular^31,32^ and square ring-shaped resonators^33,34^ have been designed. It is worth mentioning that there are other topologies such as plasmonic DEMUXes based on rectangular resonators (RRs)^35,36^, H-shaped resonators^37^, directional coupler structures^38^, and so on.

In all of the aforementioned DEMUXes, by coupling resonators with different dimensions to a central waveguide, multi-channel DEMUXes have been formed. In other words, the resonators’ sizes have been increased to obtain higher resonance wavelengths. This method paves the way for increasing the designed structures’ dimensions. Since one of the main features to design optical devices is their compact size, it is important to decrease their size as much as possible.

In this paper, a novel technique has been used to design plasmonic DEMUXes with two, four, and six channels^39,40^. The proposed structures consist of symmetrical RRs containing silver nano-rod defects (NRDs). In this method, the resonance wavelengths of different output channels have been moved to the higher wavelengths without needing to increase the resonators’ sizes. In other words, by changing the defect numbers or their radii, various resonance modes at the output channels of the plasmonic DEMUXes have been obtained. Accordingly, this technique aims to reduce the footprints of the proposed DEMUXes. Based on the obtained results, a reconfigurable structure can be created by increasing extra output channels into the basic structure. In other words, the reconfigurability of the proposed structure shows that the number of the output channels can be changed without disturbing the output transmission spectra. The metal material of the substrate area is assumed to be silver, which is characterized by the well-known Drude model^41,42^. Meanwhile, the insulator material used to fill the structures is air. The FDTD method has been used for the numerical investigation of the designed topologies.

The following sections are organized as follows: “Basic filter design” introduces the basic filter structure which is used to design the proposed DEMUXes. The proposed 1 × 2, 1 × 4, and 1 × 6 plasmonic DEMUXes have been presented in “Two-channel DEMUX design”, “Four-channel DEMUX design” and “Six-channel DEMUX design”, respectively. “Discussions and comparisons” summarizes the results and also compares them with some recently published works. Finally, the last section is devoted to “Conclusions”.

## Basic filter design

**Figure 1:**
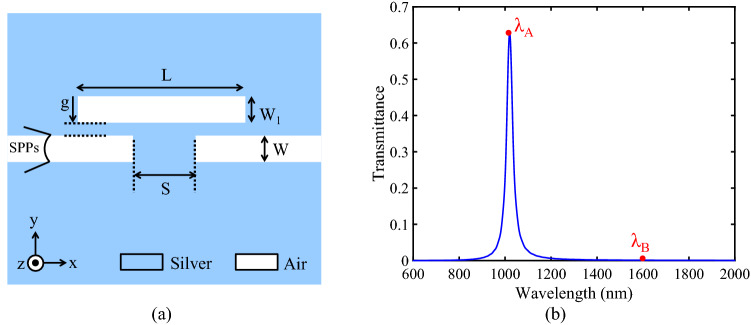
**(a)** Schematic of the basic filter. **(b)** Its transmission spectrum.

Figure 1a shows the basic filter structure. This structure consists of a RR side-coupled to two MIM waveguides. RRs are among the most extensively used resonator structures for the realization of many various types of plasmonic devices due to their key advantages. These benefits include their easy implementation, good coupling ability with MIM waveguides, and two design structural parameters (length and width of the RR) which allow the designers to achieve optimal structures. Accordingly, the RR has been selected to design the proposed structures in this paper. The structural parameters of the proposed basic filter in Fig. 1a are as follows: the length of the RR (L = 308 nm), the width of the RR (W_1_ = 50 nm), the widths of the MIM waveguides (W = 50 nm), the coupling distances between the waveguides and the RR (g = 20 nm), and the space between the two MIM waveguides (S = 140 nm). The insulator material in the white areas is air and the metal material in the blue areas is silver. The relative permittivity of silver is characterized by the well-known Drude model^43^:

1 $${{\varepsilon }}_{\mathrm{m}}\left({\omega }\right)={{\varepsilon }}_{{\infty }}-\frac{{{\omega }}_{\mathrm{p}}^{2}}{{\omega }\left({\omega }+\mathrm{j}\,\mathrm{\gamma}\right)}$$

In this formula, ε_∞_ is the medium dielectric constant for the infinite frequency, ω_p_ is the bulk plasma frequency, ɤ is the electron collision frequency, and ω is the angular frequency of incident light. The values of the mentioned parameters for silver are ε_∞_ = 3.7, ω_p_ = 1.38 × 1016 Hz, and ɤ = 2.73 × 1013 Hz. The FDTD simulation transmission spectrum of the basic filter is shown in Fig. 1b. As seen in this figure, the basic filter generates a single mode at the resonance wavelength of 1020 nm with a maximum transmission peak of 62.6%.

**Figure 2:**
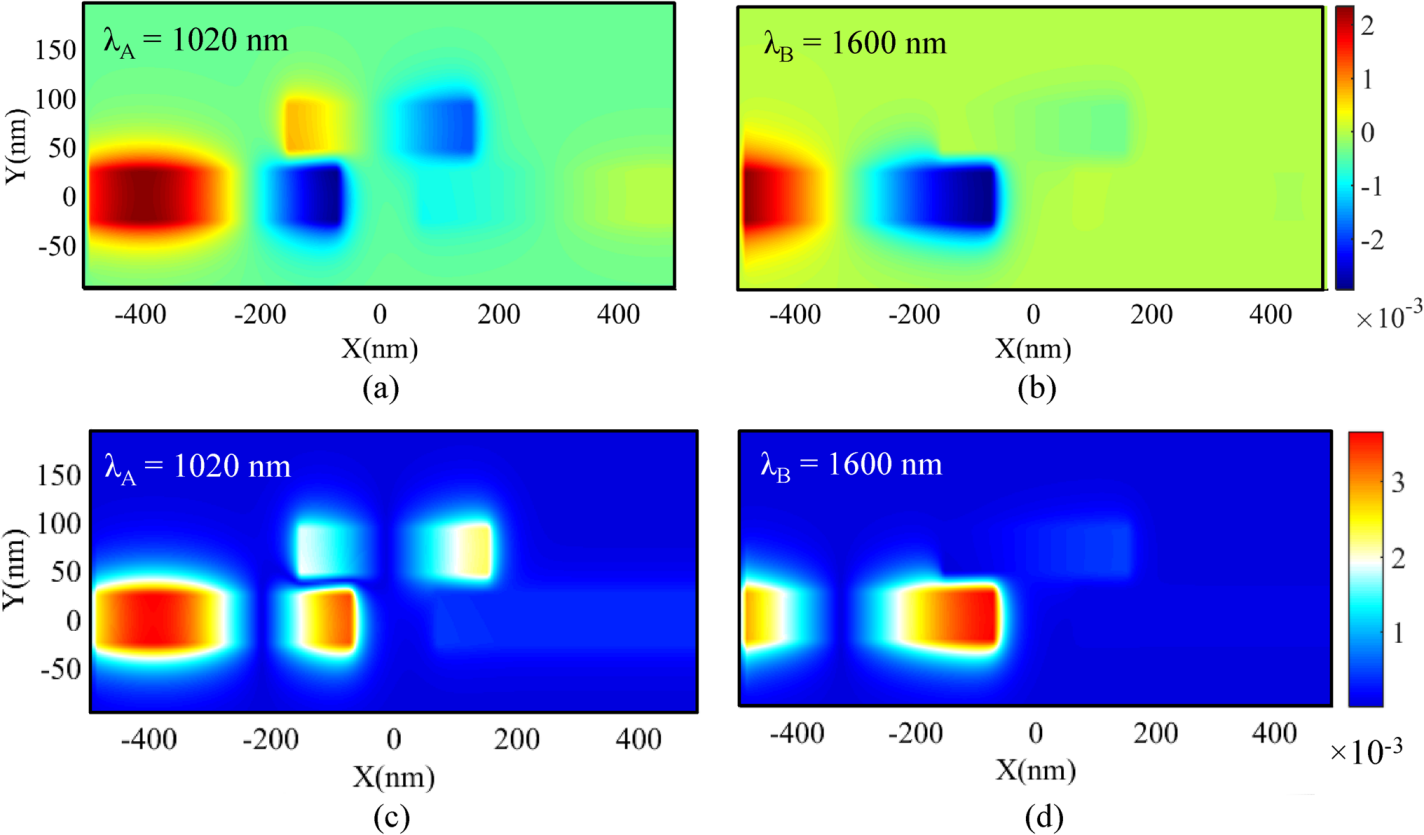
Field profile of Re (Hz) at **(a)** resonance wavelength. **(b)** Non-resonance wavelength. Field profile of $\left|{\mathrm{H}}_{\mathrm{z}}\right|$ at **(c)** resonance wavelength. **(d)** Non-resonance wavelength.

To give a better view of the basic filter’s performance, the field profiles of Re (Hz) and $\left|{\mathrm{H}}_{\mathrm{z}}\right|$ for two resonance and non-resonance wavelengths (λ_A_ = 1020 and λ_B_ = 1600 nm) have been presented in Fig. 2. As seen in Fig. 2a,c, the resonance wavelength of λ_A_ has appeared in the RR and can pass through the structure. Also, Fig. 2b,c show that the non-resonance wavelength of λ_B_ cannot direct to the output port.

**Figure 3:**
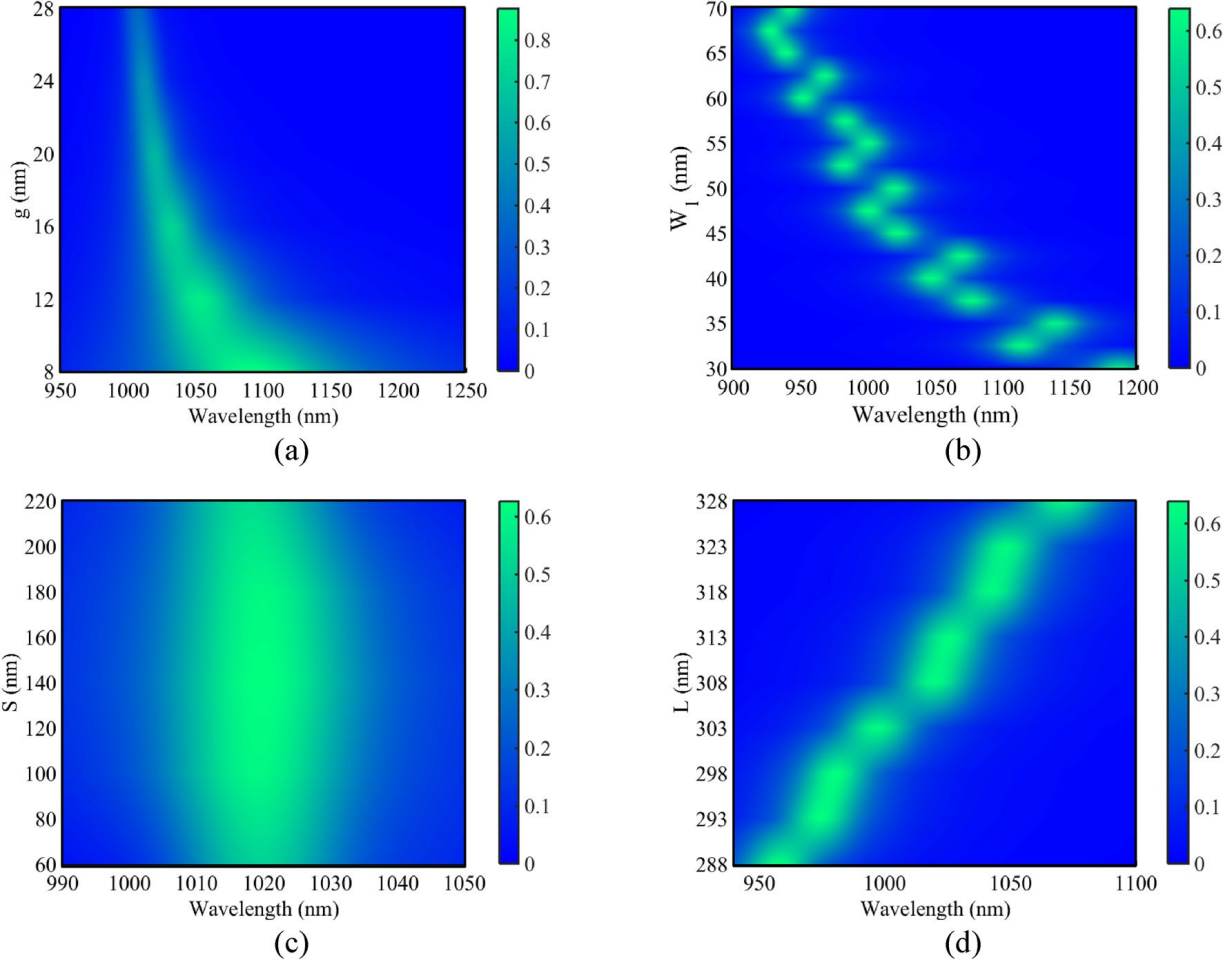
Transmission spectra of Fig. 1a for different values of **(a)** g, **(b)** W_1_, **(c)** S, **(d)** L.

After designing the basic filter, to consider the effect of the structural parameters on the transmission spectrum, the parameters values have been swept. Figure 3 shows the transmission spectra of the basic filter for different values of g, W_1_, S, and L. At first, the transmission spectrum as a function of g has been studied. As seen in Fig. 3a, by increasing the g value from 8 to 28 nm, the maximum transmission peak and the full width at half maximums (FWHM) of the resonance mode decrease. This is because by increasing the coupling space between the RR and MIM waveguides (g), the coupling strength between them decreases.

Figure 3b shows the transmission spectrum of the basic filter as a function of the width of the RR (W_1_). Here, W_1_ has been increased from 30 to 70 nm and the other parameters remain constant. As seen, increasing W_1_ corresponds to lower resonance wavelengths. The other parameter is S (Fig. 3c). As seen, when the value of S is increased from 60 to 220 nm, the maximum transmission peak and the FWHM of the resonance mode increase initially and then decrease. The last parameter is the length of the RR (L). Figure 3d shows this case. As seen in this figure, by increasing the L value from 288 to 328 nm, the maximum transmission peak increases. Accordingly, the resonance wavelength of the basic filter can be easily tuned by increasing L.

**Figure 4:**
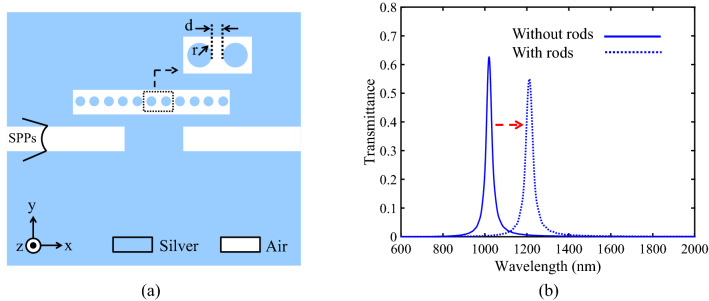
**(a)** Schematic of the basic filter with silver NRDs. **(b)** Comparison of the transmission spectrum of the basic filter with and without NRDs.

In this paper, for shifting the resonance wavelength of the designed basic filter without needing to increase the resonator size, the proposed method in^40^ has been used. Therefore, the basic filter with NRDs in the RR has been considered as follows. Figure 4a shows the basic filter with silver NRDs. The structural parameters of the NRDs are the radius of the silver nano-rods (r = 9 nm) and the distance between the nano-rods (d = 10 nm).

The proposed resonator structure can be considered as a 2D photonic crystal for SPPs, a propagating surface electromagnetic wave on a metal–insulator interface. This periodic optical structure in insulator and metallic media is named plasmonic crystal^44^. Such structures generate significant interest due to their potential for optical device miniaturization. Accordingly, different sub-wavelength devices based on plasmonic crystal structures have been designed and fabricated so far^45–48^. It should be noted that the fabrication process of the proposed structures is similar to what is discussed in^45–48^.

One of the main features of optical devices is their footprint area, which is considered in this paper. Using NRDs inside the RR (plasmonic crystal structure) leads to the size reduction in plasmonic devices. In addition to the footprint issue, using NRDs has also another benefit. The most amazing feature of this method is having a tunable resonance wavelength so that by changing the size or number of the NRDs, the resonance wavelength can be freely adjusted. Based on the mentioned advantages, such structures are suitable choices for the realization of DEMUX structures.

The transmission spectra of the basic filter with and without NRDs are compared in Fig. 4b. Figure 4b shows that, by adding the silver NRDs to the basic filter, the transmission spectrum shifts to a higher wavelength without a significant reduction in the maximum transmission peak.

As seen in Fig. 4b, the transmission bands of the basic structures (with and without NRDs) are relatively narrow for the present two-dimensional FDTD simulations. It is because there is no field radiation in this case and the field is completely confined to the waveguides and RR. In^49^, a slit-type filter that is similar to the proposed basic filter has been simulated using the 3D FDTD method and the obtained results have been compared with the 2D model. According to the presented results in^49^, undesirable transmission appears at the high wavelengths for the 3D model. This undesirable transmission is due to the radiation fields occurring at the discontinuity of the waveguide are coupled to the output port. Fortunately, a suitable procedure has been proposed in^49^ to suppress the undesirable transmission at the higher wavelengths and achieve a narrow bandwidth spectrum in the 3D model. Therefore, the proposed approach in^49^ can also be used for the 3D analysis of the proposed structures in this paper.

**Figure 5:**
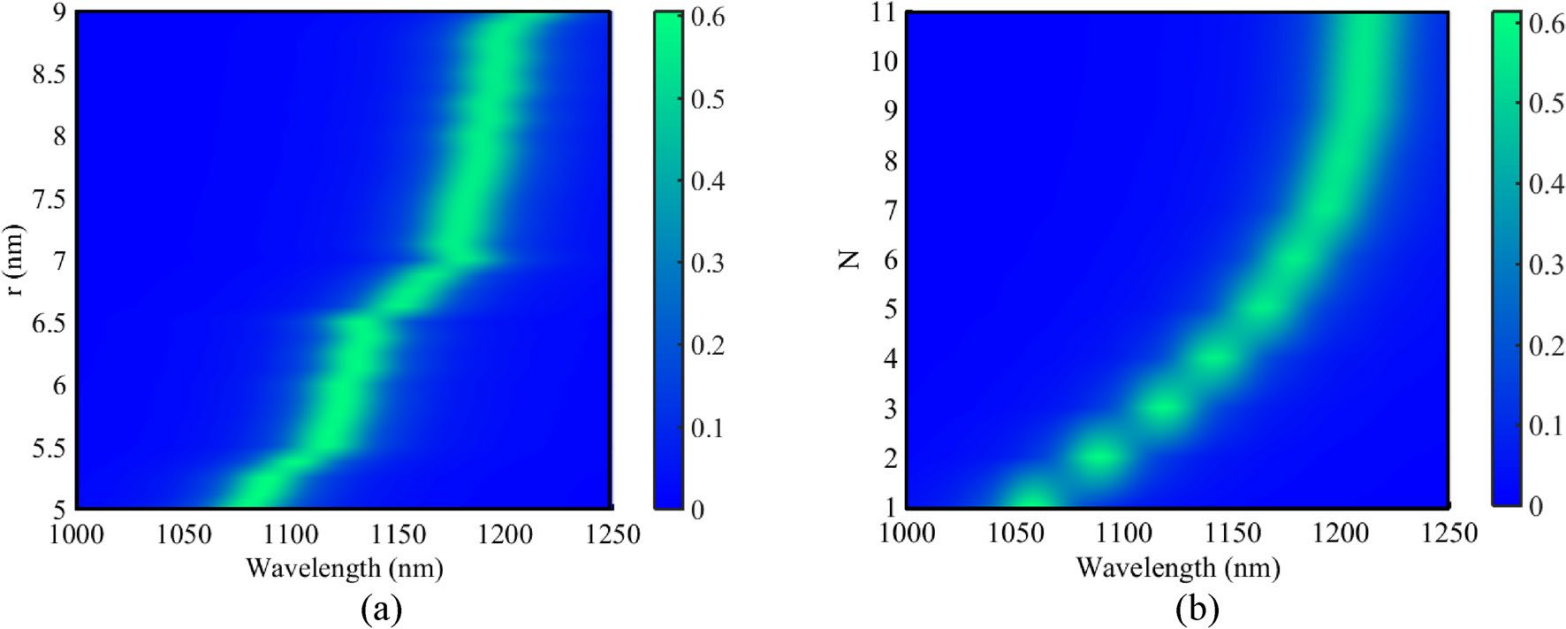
Transmission spectra of Fig. 4a for different values of **(a)** r, **(b)** N.

To study the effect of the nano-rods structural parameters on the transmission spectrum of the basic filter containing the NRDs, the parameters’ values of r (radius of the silver nano-rods) and N (number of the defects) have been swept. These variations are shown in Fig. 5. As seen, increasing the radius of the silver nano-rods and the number of the defects correspond to higher wavelengths. As a result, by coupling the basic filter with various radii and numbers of NRDs in the RRs to a central waveguide, multi-channel DEMUXes can be designed. The design procedure of the proposed DEMUXes is investigated in the next sections.

## Two-channel DEMUX design

**Figure 6:**
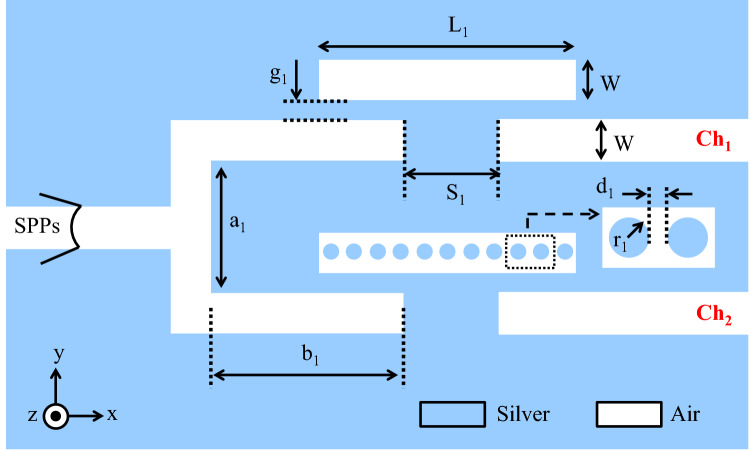
Schematic of the proposed two-channel DEMUX.

As known, single-mode filters are much more helpful to design more complex structures such as optical DEMUXes. As a result, by employing the proposed single-mode basic filter in the previous section, a two-channel plasmonic DEMUX has been designed in this section. Figure 6 shows the schematic of the proposed 1 × 2 plasmonic DEMUX. The structural parameters of the two-channel DEMUX are as follows: L_1_ = 330, W = 50, g_1_ = 20, S_1_ = 140, a_1_ = 210, b_1_ = 300, r_1_ = 10, and d_1_ = 10 (all in nm). As seen, resonators of the same size are used within each branch of the proposed two-channel DEMUX. As discussed, by introducing the NRDs in the RRs, the resonance mode can shift to the higher wavelengths. Accordingly, to obtain different resonance wavelengths at the output ports, silver NRDs are embedded in one of the RRs.

**Figure 7:**
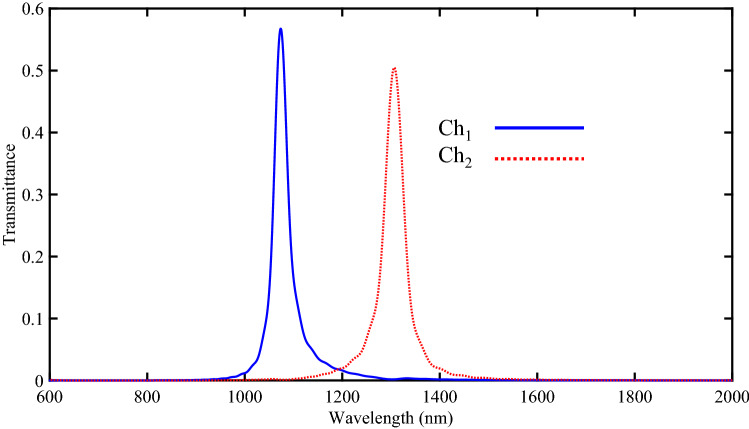
Transmission spectra of the proposed two-channel DEMUX. Also, the field profiles of Hz magnitude for two output channels of the proposed DEMUX are shown in Fig. 8. It should be noted that in Fig. 8a the resonance wavelength of 1074 nm is coupled to the upper RR (RR without the silver NRDs). Also, Fig. 8b shows that the resonance wavelength of 1307 nm appears in the lower RR (RR with the silver NRDs).

The transmission spectra of the proposed DEMUX can be calculated using the FDTD method. Figure 7 shows the transmission spectra of two output channels of the proposed 1 × 2 DEMUX. This figure shows that the selected wavelengths for Ch_1_ and Ch_2_ are 1074 and 1307 nm with the maximum transmission peaks of 56.7% and 50.5%, respectively.

**Figure 8:**
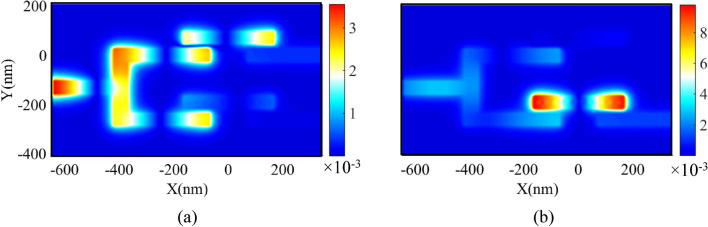
Field profile of $\left|{\mathrm{H}}_{\mathrm{z}}\right|$ for 1 × 2 DEMUX at the wavelength of **(a)** 1074 and **(b)** 1307 nm (Visual 1 and Visual 2).

## Four-channel DEMUX design

**Figure 9:**
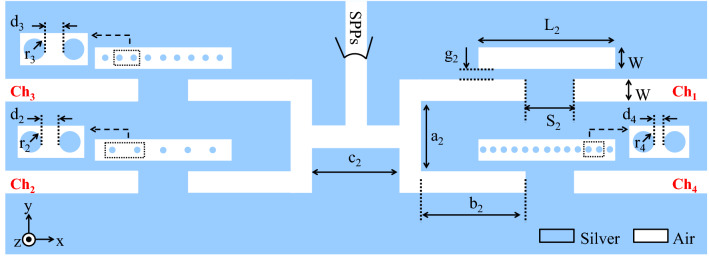
Schematic of the proposed four-channel DEMUX.

By using the same method reported in the previous section, a four-channel plasmonic DEMUX using the basic filter is proposed in this section. Figure 9 shows the schematic of the designed 1 × 4 plasmonic DEMUX. The structural parameters of the proposed DEMUX are chosen as: L_2_ = 400, W = 50, g_2_ = 10, S_2_ = 50, a_2_ = 210, b_2_ = 325, c_2_ = 300, r_2_ = 11, d_2_ = 38, r_3_ = 11, d_3_ = 23, r_4_ = 11, and d_4_ = 8 (all in nm). Similar to the two-channel DEMUX, by employing different numbers of the silver NRDs in the RRs, various resonance wavelengths at the output ports have been obtained.

**Figure 10:**
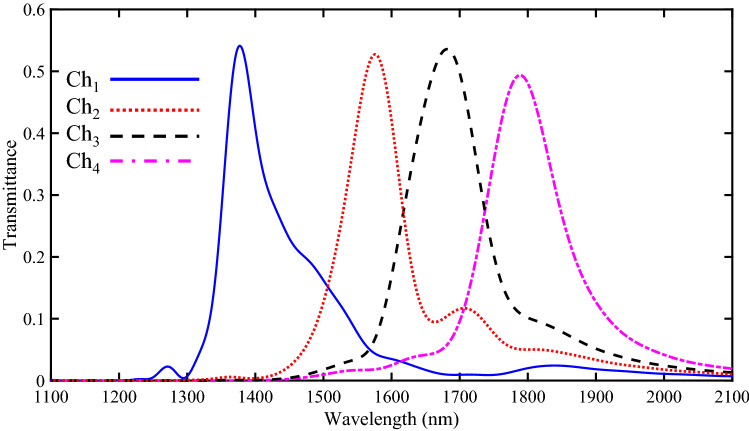
Transmission spectra of the proposed four-channel DEMUX.

The transmission spectra of the designed four-channel DEMUX using the FDTD method are shown in Fig. 10. The transmitted resonance wavelengths for Ch_1_, Ch_2_, Ch_3_, and Ch_4_ are 1377, 1576, 1682, and 1789 nm, respectively. Also, the maximum transmission peaks of these four channels are 54.13%, 52.75%, 53.6%, and 49.4%, respectively.

**Figure 11:**
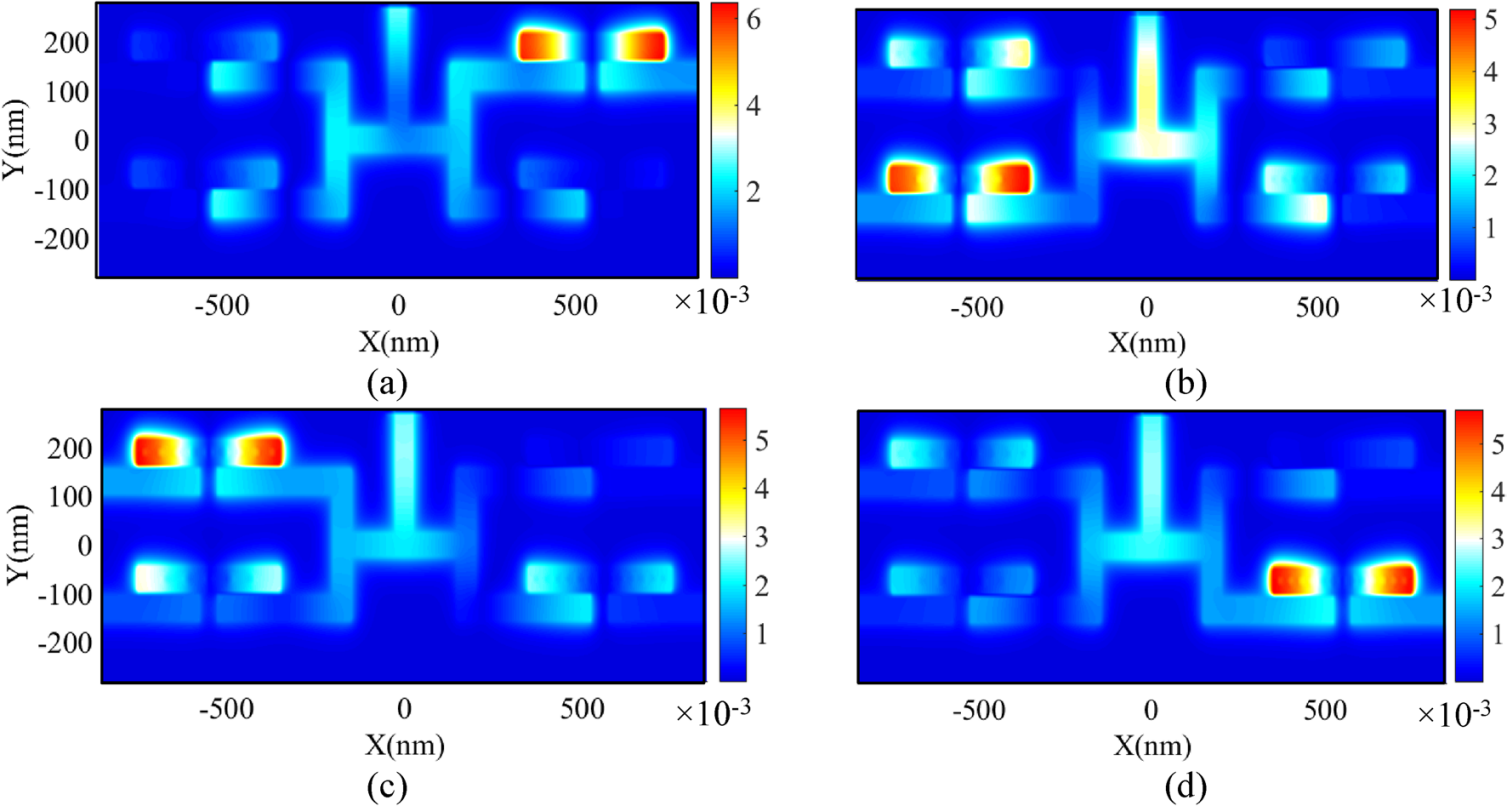
Field profile of $\left|{\mathrm{H}}_{\mathrm{z}}\right|$ for 1 × 4 DEMUX at the wavelength of **(a)** 1377, (b) 1576, **(c)** 1682 and **(d)** 1789 nm.

To provide a better view of the wavelength demultiplexing operation, the field profile of Hz magnitude for the proposed DEMUX has also been presented. The obtained results are shown in Fig. 11. As seen, the incident lights at the wavelengths of 1377, 1576, 1682, and 1789 nm can pass through the resonators of Ch_1_, Ch_2_, Ch_3_, and Ch_4_, respectively.

## Six-channel DEMUX design

**Figure 12:**
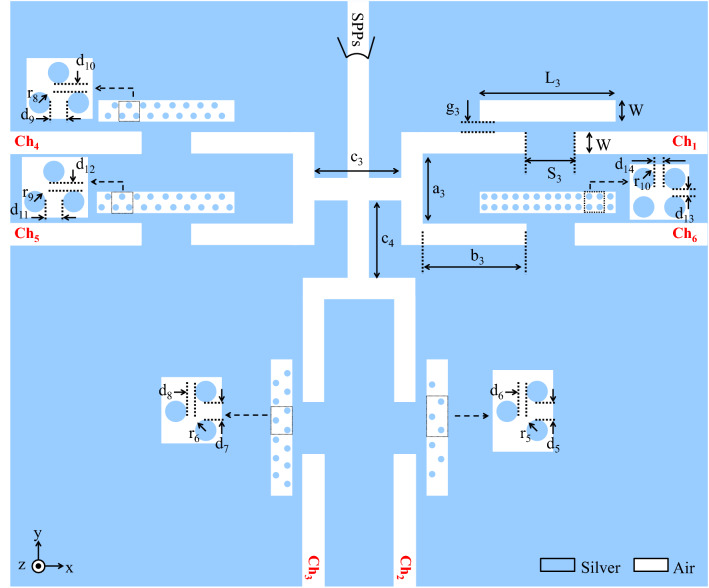
Schematic of the proposed six-channel DEMUX.

Another plasmonic DEMUX which is designed based on the proposed basic filter is a 1 × 6 DEMUX. Figure 12 shows the proposed structure. The chosen structural parameters of the six-channel DEMUX are L_3_ = 400, W = 50, g_3_ = 10, S_3_ = 50, a_3_ = 210, b_3_ = 325, c_3_ = 300, c_4_ = 200, r_5_ = 9, d_5_ = 52, d_6_ = 6, r_6_ = 9, d_7_ = 42, d_8_ = 6, r_7_ = 6.5, d_9_ = 32, d_10_ = 11, r_8_ = 7, d_11_ = 26, d_12_ = 10, r_9_ = 7.5, d_13_ = 9, and d_14_ = 15 (all in nm).

**Figure 13:**
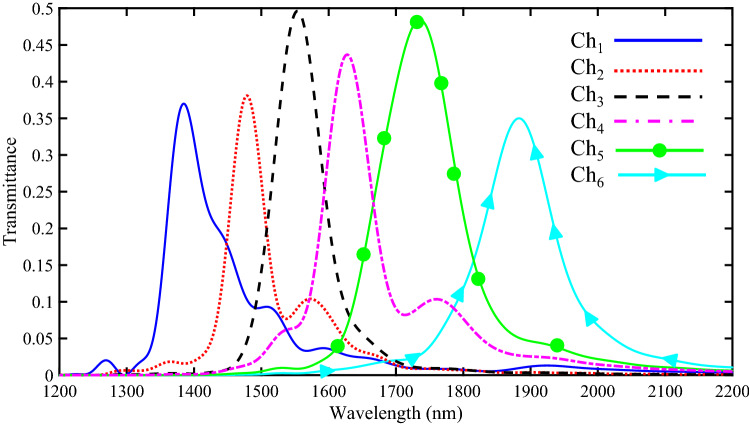
Transmission spectra of the proposed six-channel DEMUX.

Figure 13 shows the transmission spectra of six output channels. Since NRDs with different radii and numbers have been used in the RRs, there are six various transmitted resonance wavelengths for different output channels. The resonance wavelengths of six output channels (Ch_1_ to Ch_6_) are 1385, 1479, 1553, 1628, 1736, and 1883 nm with maximum transmission peaks of 37%, 37.15%, 49.62%, 43.67%, 48.43%, and 35%, respectively.

**Figure 14:**
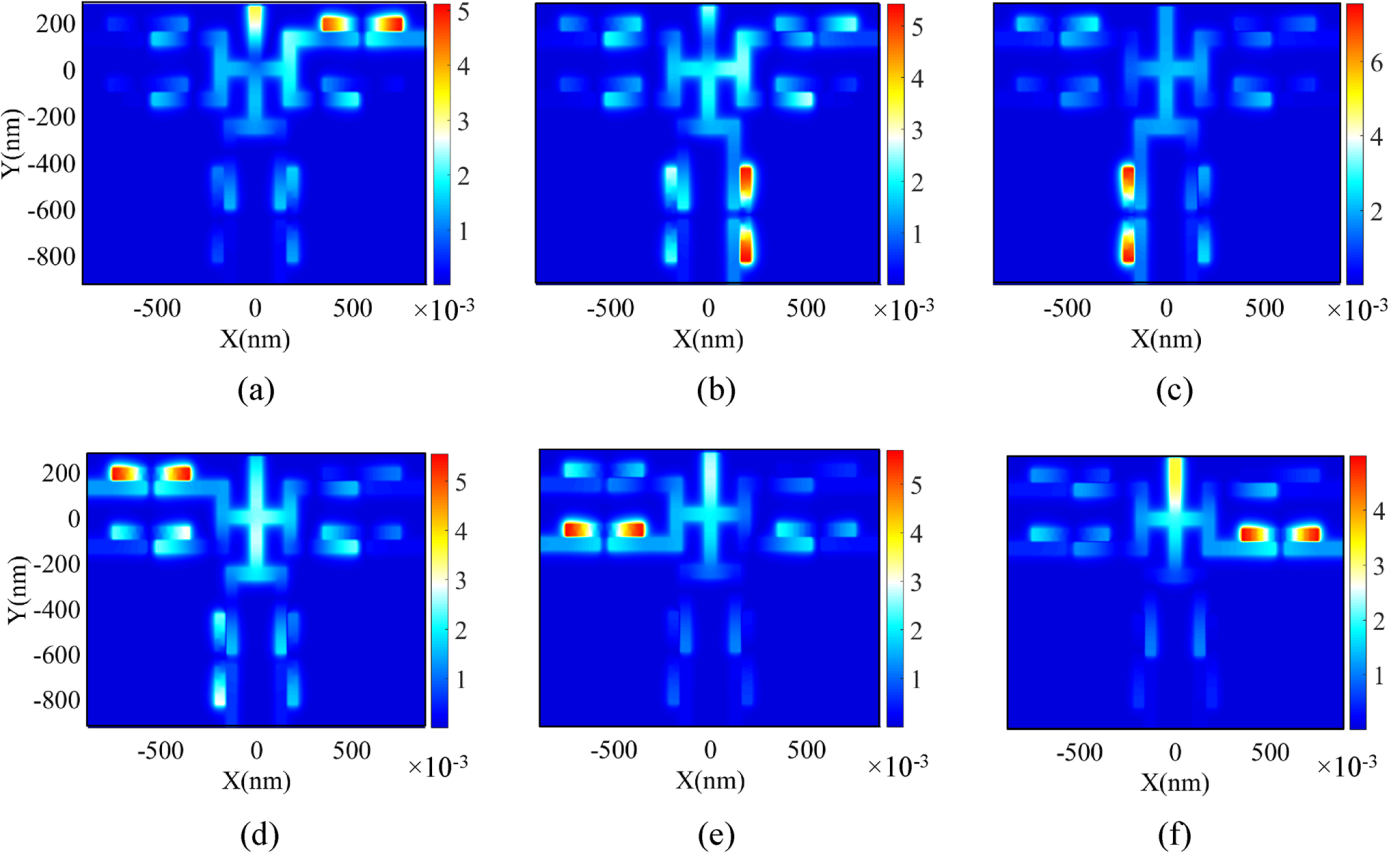
Field profile of $\left|{\mathrm{H}}_{\mathrm{z}}\right|$ for 1 × 6 DEMUX at the wavelength of **(a)** 1385, **(b)** 1479, **(c)** 1553, **(d)** 1628, **(e)** 1736, **(f)** 1883 nm.

The field profile of H_Z_ magnetic for six-channel DEMUX is also presented in Fig. 14. As seen, the wavelengths of 1385, 1479, 1553, 1628, 1736, and 1883 nm appear in the first to sixth RRs, respectively.

As seen, the crosstalk characteristics in Figs. 10 and 13 (four-channel and six-channel DEMUXes) are not as good as Fig. 7 (two-channel DEMUX). In other words, by increasing the number of the output channels, the channel spacings of the proposed DEMUXes decrease which is a common issue. As discussed, the RRs with the same dimensions incorporating different numbers and radii of NRDs have been used to design plasmonic DEMUXes in this paper. As a result, an idea can be proposed to improve the crosstalk characteristics of the presented structures when a DEMUX structure with higher channel spacing values is needed. The proposed idea is that in addition to inserting NRDs inside the resonators, RRs with various dimensions for different channels have been used. Using such a method causes that the output resonance wavelengths can be tuned over a wider wavelength range. To optimize of the structure several advanced methods are proposed^50–57^. For example, learning machine, boosted binary, and colony approaches are in spotlight of optimization methods^58–63^. More recently, whale optimization methods^64^, moth flame optimizer^65^, grasshopper optimizer^66^,, grey wolf optimizer^67^, wolf method^68^, and fruit fly optimizers^69^ are considered.

The general structures for two, four, and six-channel DEMUXes using the proposed method have been presented. Based on the potential applications of the proposed method, the presented topologies can be redesigned for other desired and applied output wavelengths including the CWDM wavelengths by changing the dimensions of RRs, NRDs and number of NRDs. This platform can be used as a main components of practical devices such as nanofluid^70–75^, BP nanosheets/Polyurethane^76^, sensorless^77^, carbon-fiber/semimetal Bi nanosheet arrays^78^, nanobeam^79^, and nanostructure devices^80–85^.

## Discussions and comparisons

As mentioned, the proposed structures are simulated using FDTD method. The “Lumerical’s FDTD solutions” software is used for this purpose. The mesh sizes that have been used in FDTD simulations are Δx = Δy = 2 nm. Furthermore, the perfectly matched layer (PML) by a thickness of 200 layers is considered as the boundary condition^86^.

To provide a better view of the obtained results, the proposed DEMUXes have been compared with other reported works in the literature. Table 1 shows some main features of DEMUXes for comparison. The comparison parameters include the metal model, topology of the designed structures, number of output channels (N), resonance wavelengths of output channels (λ_r_) with their transmittance values (T), FWHM and quality factor (Q-factor) of the resonance wavelengths, the average of the channel spacings for each DEMUX, and DEMUX sizes.

**Table 1:** Performance comparisons between the proposed DEMUXes and other works.

Ref	Metal model	Topology	N	λ_r_ (nm)	T (%)	FWHM (nm)	Q-factor	Channel spacing (nm)	Size (μm^2^)
Ref^16^	Drude	Improved disk resonator	3	1204 1267 1372	46 40 50	36 38 36	33.44 33.34 38.11	84	1.984
Ref^17^	Drude	Ring-shaped resonator	2	1310 1530	80 99	342.04 434.66	3.83 3.52	220	1.359
Ref^28^	Drude	Disk resonator	3	1310 1430 1550	55.26 57.3 56.9	99.85 40.7 38.85	13.12 35.13 39.9	120	4.727
Ref^32^ 1st	Drude	Ring-shaped resonator	2	1310 1550	46 45	58.85 90.11	22.26 17.2	220	1.05
Ref^32^ 2nd	Drude	Ring-shaped resonator with metallic slit	2	1310 1550	34.5 19.6	62.38 99.35	21 15.6	220	1.05
Ref^37^ 1st	Drude	H-shaped resonator	2	1435 1485	98.7 94.3	35 40	47.8 37.125	50	0.37
Ref^37^ 2nd	Drude	H-shaped resonator	4	1435 1485 1550 1615	98.7 94.3 94.2 96.1	35 37.98 62.32 79.5	41 39.1 24.87 20.31	60	0.832
This work (1 × 2)	Drude	RR & silver nano-rods	2	1074 1307	56.7 50.5	32.56 44.95	33 29.1	233	0.285
This work (1 × 4)	Drude	RR & silver nano-rods	4	1377 1576 1682 1789	54.13 52.75 53.6 49.4	83.1 91.1 121.4 122.5	16.57 17.3 13.86 14.6	137.33	0.368
This work (1 × 6)	Drude	RR & silver nano-rods	6	1385 1479 1553 1628 1736 1883	37 37.15 49.62 43.67 48.43 35	88.4 57 80.2 78.8 127.5 120	15.67 25.94 19.36 20.66 13.62 15.7	99.6	1.87

As seen in Table 1, all of the reported works have two or three output channels except for the four-channel DEMUX reported in^37^, while the reported structure can be extended to a six-channel DEMUX. Furthermore, in terms of other comparison parameters such as the transmittance values of the resonance wavelengths, FWHM, Q-factor, and channel spacing value, the designed structures have relatively better characteristics among the quoted DEMUXes.

Here, each of the mentioned parameters is separately studied. An ideal DEMUX should be able to pass resonance wavelengths without any weakening. In other words, the transmittance values of the resonance wavelengths should be high. Since plasmonic structures are inherently lossy, the maximum transmission values of plasmonic DEMUXes (especially in DEMUXes with more output channel numbers) cannot increase dramatically. As seen in Table 1, the transmittance values of the designed DEMUXes (considering the six-channel DEMUX) are suitable among the reported works.

The other two important features for designing plasmonic DEMUXes are FWHM and Q-factor. As known, a lower FWHM in a resonance mode results in a higher Q-factor. As seen in the comparison table, the proposed structures have medium Q-factor values. It is worth mentioning that although using high Q-factor resonators creates high distinctions between the different channels in a plasmonic DEMUX, but fabrication of ultra-high Q-factor structures has its own predicaments.

The channel spacing parameter in Table 1 shows the average of the channel spacings for each DEMUX. A higher channel spacing value in a DEMUX shows a higher distinction between its output resonance wavelengths. As seen, channel spacing values of all three designed structures are high. It is worth mentioning that the proposed two-channel DEMUX has the highest channel spacing value among the reported references^87,88^.

As mentioned, all of the reported DEMUX structures use a resonator type with various dimensions for different channels. This technique causes increasing the footprint areas of the DEMUX structures. Another most outstanding feature of the proposed novel method to design the presented DEMUXes in this paper is that different resonance wavelengths for output channels can be obtained without any increment in the resonator size. As a result, the proposed structures are compact DEMUXes compared to other topologies.

## Conclusion

In this paper, plasmonic wavelength DEMUXes with two, four, and six output channels were proposed. The designed structures are composed of symmetrical RRs containing silver NRDs and MIM waveguides. The FDTD simulation results indicate that by varying the silver nano-rods’ numbers and radii, different output wavelengths can be obtained at the output channels of DEMUXes. According to the simulation results, for two, four, and six-channel DEMUXes, the maximum transmission values of 56.7%, 54.13%, and 49.62% and the average channel spacing values of 233, 137.33, and 99.6 nm have been obtained, respectively. The simple and compact designed DEMUX structures are promised for integrated optical circuits.

## Data Availability

The calculated results during the current study are available from the corresponding author on reasonable request.
